# Microbiological and Chemical Properties of Chokeberry Juice Fermented by Novel Lactic Acid Bacteria with Potential Probiotic Properties during Fermentation at 4 °C for 4 Weeks

**DOI:** 10.3390/foods10040768

**Published:** 2021-04-03

**Authors:** Christos Bontsidis, Athanasios Mallouchos, Antonia Terpou, Anastasios Nikolaou, Georgia Batra, Ioanna Mantzourani, Athanasios Alexopoulos, Stavros Plessas

**Affiliations:** 1 Laboratory of Food Processing, Faculty of Agriculture Development, Democritus University of Thrace, 68200 Orestiada, Greece; bontsas@gmail.com (C.B.); imantzou@agro.duth.gr (I.M.); alexopo@agro.duth.gr (A.A.); 2 Laboratory of Food Chemistry and Analysis, Department of Food Science and Human Nutrition, Agricultural University of Athens, 11855 Athens, Greece; amallouchos@aua.gr; 3 Department of Agricultural Development, Agri-Food, and Natural Resources Management, School of Agricultural Development, Nutrition & Sustainability, National and Kapodistrian University of Athens, 34400 Psachna, Greece; aterpou@agro.uoa.gr; 4 Laboratory of Applied Microbiology & Biotechnology, Department of Molecular Biology & Genetics Health Sciences Faculty, Democritus University of Thrace, Building #10, University Campus, 68100 Alexandroupolis, Greece; anikol@mbg.duth.gr; 5 Department of Food Science & Nutrition, School of Agricultural Sciences, University of Thessaly, 43100 Karditsa, Greece; gogo.batra@gmail.com

**Keywords:** chokeberries juice, *Aronia melanocarpa*, *Lactobacillus paracasei* SP5, antioxidant activity, phenolics, volatiles, functional beverage

## Abstract

On the frame of this research survey, a novel potentially probiotic strain (*Lactobacillus paracasei* SP5) recently isolated from kefir grains was evaluated for chokeberry juice fermentation. Chokeberry juice was retrieved from the variety *Aronia melanocarpa*, a plant known to provide small, dark berries and to be one of the richest sources of antioxidants. The juice was subsequently fermented inoculating *L. paracasei* SP5 for 48 h at 30 °C. The fermented juices were left at 4 °C and tested regarding microbiological and physicochemical characteristics for 4 weeks. The potentially probiotic strain was proved capable of performing lactic acid fermentation at 30 °C. Cell viability of *L. paracasei* was detected in high levels during fermentation and the whole storage period, while the fermented juice showed higher levels of viability in juice with 40.3 g/L of initial sugar concentration. No ethanol was detected in the final fermented juice. Fermented chokeberry juice was characterized by aromatic desirable volatiles, which were retained in adequate levels for the whole storage period. Specifically, the occurrence of organic esters detected in fermented juices is considered as positive evidence of the provision of fruity and floral notes to the final product. During storage, total phenolics content and antioxidant activity were observed in higher levels in fermented chokeberry juice compared with non-fermented juice. Subsequently, fermentation of chokeberry juice by potentially probiotic lactic acid bacteria could provide high industrialization potential, providing the market with a nutritional beverage of good volatile quality with an enhanced shelf-life compared with an unfermented fresh juice.

## 1. Introduction

Fruit berries have received worldwide attention and are being promoted as part of the human diet due to their nutritional and bioactive value. Many research papers in the literature evince the significant health effects that berries pose [1]. These health effects are mainly provided due to their high nutrient content, including vitamins, minerals, proteins, and polyphenols [1,2]. Black chokeberry or aronia berries (*Aronia melanocarpa*) contain high polyphenol levels, providing significant antioxidant capacity [3]. Due to their high amount of antioxidant molecules, chokeberries exhibit various health effects, including antidiabetic, antioxidative, cardioprotective, hepatoprotective, anticancer properties, and even antibacterial properties [3,4,5]. However, chokeberries display a significant drawback related to the organoleptic properties of their juice, which frustrates consumers. Specifically, chokeberries are described as astringent, sour, and bitter due to their high procyanidin content [6]. Nowadays, these purple-black berries are being used for fresh and pasteurized juice production, for fermentation in fruit wine production, and as colorants in the food industry [6]. The application of lactic acid fermentation to chokeberry juice could be an effective alternative, targeting enriched flavor and high bioactive compounds availability. This attempt seems very interesting since it fits with the modern trend in fruit juice bioprocessing [7,8,9], while recently, the application of lactic acid fermentation to pomegranate juice enhanced the flavor and shelf life of the fermented juice, ensuring a better control of the volatile characteristics and microbial population during storage [7]. A similar outcome was verified through the determination of increased desired volatile organic compounds (VOC), limiting in parallel the non-desired volatile compounds [10]. 

The multiple effects of lactic acid fermentation on the nutritional value of fruit juices has been extensively studied [7,11,12]. Lactic acid bacteria may modify the level and bioavailability of nutrients through their metabolism or through their interaction with the gut microbiota or even with the human immune system [7,13]. Indeed, several phenolic derivatives showed higher antioxidant activity than their precursors after lactic acid fermentation [10,14]. In addition to the bioconversion of phenolic compounds, lactic acid fermentation may also deliver other modifications to food ingredients, with influence on consumers health. For instance, several fruits and vegetables have been proved to exhibit higher composition of phenolic derivatives, providing in parallel high bioavailability after lactic acid fermentation with selected *Lactobacillus* spp. [14,15,16]. 

Moreover, fruit juices have gained more attention in recent years as vehicles for probiotic delivery versus dairy products because there are more and more consumers who have shifted their interest to plant products, particularly vegetarians [13,17,18]. The main reasons for this turn are lactose intolerance, high cholesterol content of meat and dairy products, and allergic effects of milk proteins, which are limiting factors in the growth of dairy- or even meat-based probiotics [19,20,21]. In the past few years fruit juices have become very popular for probiotic delivery through lactic acid fermentation, as proposed by many researchers [18,22,23]. Regarding lactic acid fermentation of chokeberry juice, few research attempts have been recorded in the literature till now. Specifically, lactic acid fermentation of chokeberry juice with four different strains of *L. plantarum* provided an alternative beverage with good polyphenols content, but without recording issues with its sensorial properties, which can be considered a significant outcome [8]. Moreover, kefir was applied in chokeberry juice fermentation resulting in increased polyphenols bioavailability as well as increased antioxidant capacity [24].

Likewise, in the frame of this research the employment of a novel potentially probiotic strain *Lactobacillus paracasei* SP5 (now classified as *Lacticaseibacillus paracasei*) recently isolated from kefir grains [25] was studied in chokeberry juice fermentation. The main targets of the current research include: (i) the impact of lactic acid fermentation on the volatile profile of the produced fermented juice at 4 °C, (ii) the examination of chokeberry juice as a suitable substrate for probiotic delivery, and (iii) the production of a novel food product with probiotic properties that can retain acceptable physicochemical characteristics over 4 weeks at 4 °C. The viability of the potential probiotic strain, as well as the concentration of residual sugars, volatile compounds, phenolics content, antioxidant activity, and organic acids were monitored after fermentation of chokeberry juice at 30 °C and at 4 °C for 4 weeks. 

## 2. Materials and Methods

### 2.1. Microbial Culture 

The novel potential probiotic strain *Lactobacillus paracasei* SP5 was applied as starter culture for chokeberry juice fermentation. The potentially probiotic bacterial strain, recently isolated from kefir grains [25], was collected for deep cold storage (−80 °C) and activated by incubation in 10 mL of sterile skim milk at 37 °C for approximately 1 h. Subsequently, the activated strain was grown in de Man–Rogosa–Sharpe (MRS) liquid broth (Fluka, Buchs, Switzerland) at 37 °C for 24–48 h under anaerobic conditions. The biomass of *L. paracasei* SP5 was harvested by centrifugation at 5000 rpm for 10 min at 25 °C (Sigma 3K12, Bioblock Scientific, Saint Nom, France). All media were sterilized prior to use by autoclave at 120 °C for 15 min (1–1.5 atm).

The harvested biomass was submitted to freeze-drying. The harvested microbial culture of *L. paracasei* SP5 was initially frozen to −43 °C in a Biocool Controlled Rate Freezer (SP Scientific, Warminster, PA, USA) with a freezing rate of 5 °C/min. Then the frozen culture was moved aseptically on a freeze dry system, FreeZone 4.5 (Labconco, Ft. Scott, KS, Kansas, USA), and was submitted to freeze-drying for 48 h at −45 °C (5 × 10^−3^ mbar) [26]. Subsequently, the freeze-dried bacterial biomass was applied as starter culture for chokeberry juice fermentations. 

### 2.2. Chokeberry Juice Fermentation

Chokeberries were obtained from the area of Orestiada (Greece) by a local organic farming producer. The variety used was *Aronia melanocarpa* and was preserved in the freezer at −20 °C. Chokeberries were thawed at 4 °C for about 18 h and were placed in a sterile beaker. The juice was aseptically obtained using sterile filter paper. The pulp was then removed using a sterile strainer, centrifuged at 30,000 rpm for 10 min for the removal of all solids. Sterilized, deionized water was added to adjust the initial sugar concentration. No sugars were added. Afterward, the juice was pasteurized (80 °C, 10 min), and 1g of freeze-dried *L. paracasei* SP5 biomass was suspended per 100mL of fermentation substrate in different initial sugar concentrations. Specifically, three values of initial sugar concentration (61.0, 40.3, and 28.8 g/L) were tested. The pH was adjusted to 4.0 in all cases through the addition of sterile NaOH N/10 and was monitored. The substrates were left undistributed to ferment at 30 °C for 48 h and then each fermented juice was stored at 4 °C for 4 weeks. 

### 2.3. Microbiological Assessment and L. paracasei Viability

Aliquots of 10 mL were retrieved from each fermented flask of chokeberry juice after thorough homogenization at various time intervals during the 4 weeks at 4 °C. Each sample was serially diluted in 90mL of sterile Ringer’s solution of one-quarter strength (Sigma-Aldrich, Darmstadt, Germany), placed aseptically in a sterile plastic bag and homogenized in a Bagmixer (400 Model VW, Interscience). Subsequently the suspension was then subjected to serial decimal dilutions of one-quarter strength Ringer’s solution and plated on selective media.

Viable cell counts of the potentially probiotic strain (*L. paracasei* SP5) were enumerated on acidified MRS agar (Merck, Darmstadt, Germany) at 37 °C for 72h under anaerobic conditions (anaerobic jar, Anerocult C, Merck, Germany). Yeasts and fungi were enumerated on potato dextrose agar (PDA) after incubation at 30 °C for 72 h. Possible coliform cell counts were examined on Violet Red Bile Agar (VRBA) after incubation at 30 °C for 24 h [27]. 

All media were prepared according to the instructions provided by the manufacturer, were sterilized (120 °C, for 15 min, 1–1.5 atm), and then cooled to 45 °C prior to use. Cell counts were expressed as log of mean colony-forming units (cfu) per mL of chokeberry juice. 

### 2.4. Chokeberries Juice Physicochemical Analysis: Residual Sugar, Ethanol, and Organic Acids Concentration

Samples were collected at various time intervals during juice fermentation (days 2, 7, 14, 21 and 28). Residual sugars (fructose and glucose), ethanol, and organic acids (malic, lactic and acetic acid) content were determined by HPLC, using a Shimadzu chromatography system (Shimadzu Corp., Duisburg, Germany). The equipment consisted also of a DGU-20A5R degassing unit, a LC-20AD pump, and a RID-10A refractive index detector. Separation of compounds was accomplished on a Nucleogel ION 300 OA (Macherey-Nagel, Düren, Germany) thermostated at 85 °C (CTO-20AC oven) using as mobile phase an aqueous solution of 0.049 N H_2_SO_4_ at 0.3 mL/min. After double filtration (0.22 μm), 20 μL was injected directly on the column [28]. Residual sugar, organic acids, and ethanol concentration were calculated using standard curves.

### 2.5. Total Phenolics Content

The method applying Folin-Ciocalteu reagent was used for the determination of total phenol content (TPC) [11]. Briefly, 20 μL of chokeberry juice was mixed with 100 μL of Folin-Ciocalteu reagent (2 M) (Merck, Darmstadt, Germany) and 1580 μL of distilled water. Then, 300 μL of sodium carbonate solution of anhydrous sodium carbonate (Na_2_CO_3_) (Merck, Darmstadt, Germany) (200 g/L) was added to the above mixture and the solution was kept for 2 h in the dark at 20–22 °C (room temperature). The resulting absorbance was determined at 765 nm in a spectrophotometer (Shimadzu UV-1700, Tokyo, Japan). Total phenolics content was expressed as mg of gallic acid equivalents (GAE) per L of chokeberry juice. 

### 2.6. Determination of Antioxidant Activity

Antioxidant activity of chokeberry juice was determined with two methods: (i) ferric reducing antioxidant power (FRAP) assay and (ii) the 2, 2′-azinobis-(3-ethylbenzothiazoline)-6-sulfonic acid (αTEAC) assay.

#### 2.6.1. FRAP Assay

FRAP assay was performed according to the method previously reported in the literature [29]. In brief, 0.4 mL of diluted juice sample was mixed with 3 mL of acidic FRAP reagent. FeSO_4_ was used as the standard. The results were expressed as μmol of Fe^2+^ 245 equivalents per liter of sample. All measurements were repeated three times.

#### 2.6.2. αTEAC Assay

2, 2′-azinobis (3-ethylbenzothiazoline-6-sulfonic acid) (ABTS•+) was prepared by the reaction of ABTS with K_2_S_2_O_4_. Samples were analyzed at five different dilutions within the linearity range of the assay, according to the method previously reported in the literature [30]. 

### 2.7. Determination of Volatile Compounds in Fermented Chokeberry Juice by SPME/GC-MS

#### 2.7.1. Sample Preparation and Sampling

An aliquot (2 mL) of chokeberry juice was pipetted into 20 mL headspace glass vial containing 1 g ammonium sulfate. Subsequently, 10 μL of an internal standard solution (1,4-dioxane 1000 mg/L) was added. The vials were sealed with crimp caps with PTFE-lined silicone septa and equilibrated for 5 min at 40 °C under stirring at 250 rpm in a water bath. The volatiles were extracted by exposing the (solid-phase microextraction) SPME fiber (DVB/CAR/PDMS, length 2 cm, Sigma Aldrich, Germany) for 30 min under the same conditions. 

#### 2.7.2. Volatiles Evaluation by GC-MS Analysis 

The volatiles of fermented chokeberry juice were determined using GC-MS analysis. In specific, the volatiles were absorbed by a fiber and were desorbed in the injection port of GCMS-QP2010 Ultra (Shimadzu Inc., Kyoto, Japan) equipped with a suitable liner (0.7 mm i.d., Sigma Aldrich) at 240 °C in split mode (split ratio 1/1) for 5 min. Each time, the fiber was retracted and conditioned for 5 min at 250 °C in the injection port of another GC, targeting the removal of any volatile residues. 

The separation of compounds was performed in a DB-Wax capillary column (30 m × 0.25 mm i.d., 0.25 μm film thickness, Agilent, Santa Clara, CA, USA). Helium was utilized as carrier gas at a constant linear velocity of 36 cm/s. The oven temperature was programmed at 40 °C for 5 min and then the temperature increased at a rate of 5 °C/min up to 180 °C. After achieving 180 °C the temperature increased at a rate of 30 °C/min up to 240 °C and held at that temperature for 5 min. 

The mass spectrometer operated in an electron ionization mode, setting the electron energy at 70 eV and 40–300 *m*/*z* mass scan range. The source and interface temperature were set at 200 °C and 240 °C, respectively. 

Identification of volatile compounds was accomplished by comparing: (i) the retention indices based on the homologous series of n-alkanes (C8-C24, Niles, IL, USA) with those of authentic compounds (when available) and those of the NIST14 library (NIST, Gaithersburg, MD, USA), (ii) MS data with those of reference compounds and by MS data obtained from NIST14 library. GCMS solution (ver. 4.30, Shimadzu, Kyoto, Japan), AMDIS (ver. 2.72, NIST) and NIST MS Search (ver. 2.2, NIST) software were used in the identification process. 

The reliability of identification was set at three different levels:

● A-level: agreement of retention index (RI) and mass spectrum (MS) with RI and MS of an authentic compound analyzed under identical experimental conditions
● B-level: agreement of RI (ΔRI < 20) and MS (match > 900)
● C-level: agreement of at least ΔRI < 20 or MS similarity match > 800

The content of individual compounds was calculated relative to the internal standard. The final content of identified compounds was expressed as μg/g of chokeberry juice. All calculations were based on peak area (from AMDIS). 

### 2.8. Statistical Analysis

All experiments were repeated three times and the results are expressed as means plus standard deviations. Microbial populations were expressed as log of colony-forming units (cfu) per mL of chokeberry juice. Multifactor analysis of variance (MF-ANOVA), with initial sugar concentration (ISC), fermentation time, and storage time as factors, was used to test for mean differences of the various analytic results (ethanol, residual sugars, lactic, and acetic acid; cell viability; concentration of volatile compounds; and TPC) at the 95% confidence level. To discriminate among the means of the various factors, Tukey’s honestly significant difference (HSD) was employed. 

## 3. Results and Discussion

### 3.1. Cell Viability

Chokeberry juice was applied as substrate in lactic acid fermentation by the potential probiotic strain *L. paracasei* SP5 for the production of a novel functional beverage. Initially, chokeberry juice was fermented for 48 h at three different initial sugar concentrations to find out the optimal sugar content for probiotic growth. The pH value of all fermented juices was monitored at the end of 48 h fermentations and during the 4 weeks (4 °C). No significant change was observed compared with the initial pH value (4.0). Specifically, the pH value varied between 4.0 ± 0.2 for all the periods mentioned, probably due to the high buffering capacity of the fermented juices, as other researchers have reported in similar experiments regarding lactic acid fermentations of various fruit juices [12].

The selected fermented chokeberry juice of initial sugar concentration 40.3 g/L was stored for 4 weeks at 4 °C. Samples were collected after 48 h of fermentation and after each week of storage (1–4 weeks) for the determination of residual sugar, volatile compounds, lactic and acetic acid contents, total phenolics count, and cell viability. 

The viability of *L. paracasei* SP5 was monitored during fermentation and storage. The fermented juice was also monitored for the occurrence of possible spoilage microorganisms. Specifically, yeast, fungi, or coliform bacteria were not detected (nonvisible colony) after juice fermentation as well as during the four weeks of cold (4 °C) storage (Table 1). 

Initially, the viability of the potentially probiotic lactic acid bacterial strain *L. paracasei* SP5 was 9.5 log cfu/mL for all the initial sugar concentrations studied. At the end of fermentation (48 h), a significant decrease was observed in the cases of ISC of 61.0 and 28.8 g/L. A possible explanation of this decrease might be that the cells during the first 48 h were in a phase of adaptation to the new environment. Specifically, various factors such as antioxidant activity or other antimicrobial agents may have caused a limiting effect in the viability of *L. paracasei* SP5 [31]. Only in the case of ISC of 40.3 g/L was this decrease not observed. It is likely, that the adjusted respective ISC led to proper levels of sugars and maybe other nutrients, that did not affect the viability of *L. paracasei* SP5. This outcome has been reported in the past and has depended on the strain applied and the fruit juice that was to be fermented. For instance, in a relevant work conducted, the viability of *L. paracasei* K5 dropped approximately 0.5-fold after fermentation of pomegranate juice at 30 °C and continued, slightly to be decreased, during its 28 days of fermentation at 4 °C [31]. In another similar work, the viability of *L. plantarum* ATCC 14,917 decreased after fermentation of pomegranate juice and suddenly increased 30% at the end of the 14th day of fermentation at 4 °C [32]. Likewise, the initial delay in the viability of microbial cultures is not a surprise when the sugar content is not in appropriate concentrations that can be utilized, while after the first shock the microbial culture is expected to overcome this issue. 

More importantly, cell viability maintained high levels throughout 3 weeks of storage (above 7.5 log cfu/mL) in all fermented juices, providing possible probiotic characteristics to the fermented juice [13]. A significant decrease was observed during the last week of storage in all the samples. However, only in the case of the fermented fruit juice with initial sugar concentration of 28.8 g/L did the viability drop at 5.1 log cfu/mL, while the other two juices contained viable cells above 6.5 log cfu/mL. These recorded viability values were detected above the limit of 6 log cfu/mL, which is the limit required for products to be characterized as probiotic [13,25]. Likewise, between the two juices with initial concentrations of sugars of 40.3 and 60.1 g/L, the second one had significantly higher probiotic load at the last week of of fermentation at 4 °C (7.9 log cfu/mL) and thus it was chosen for the rest of the experiments. 

Increased numbers of viable cells in a food system, especially during storage, is critical for candidate probiotic microorganisms [7,13]. The stability observed in the viability of *L. paracasei* SP5 during fermentation at 4 °C of chokeberry juice (with initial sugar concentration of 40.3 g/L) was quite encouraging. The preservation of cell viability was also reported in other cases during lactic acid fermentation of fruit juices, even at fermentation at 4 °C. Specifically, experiments conducted with pomegranate juice and cornelian cherry juice fermented with other candidate probiotic *L. paracasei* strains provided high viability levels during fermentation at 4 °C [7,11,12]. A possible explanation of this outcome is that these LAB strains are very tolerant and can maintain high viability levels at low temperatures [7,11,33]. Moreover, other compounds existing in fruit juices may exhibit prebiotic properties and promote the growth of LAB [13]. For instance, it has been reported that anthocyanins from black rice can provide prebiotic properties and promote the growth of on Bifidobacteria and Lactobacilli [34]. Another factor ensuring viability of *L. paracasei* SP5 in fermented chokeberry juice is its tolerance in low pH values. As reported previously, *L. paracasei* SP5 is considered a potentially probiotic strain due to its ability to succeed in various in vitro tests with low pH tolerance being a significant one. In more detail, *L. paracasei* SP5 managed to preserve its viability during exposure in a pH value of 3 [25]. As a result, *Lactobacillus paracasei* SP5 can be considered as an acid resistant strain, which makes it appropriate for fruit juice fermentation where the pH values are usually very low.

### 3.2. Ethanol, Organic Acids, and Residual Sugar Concentration

The results of sugars (glucose, fructose, sorbitol), organic acids (malic acid, lactic acid, acetic acid), and ethanol in chokeberry juice during fermentation (30 °C for 48 h) and storage (4 °C for 4 weeks) are presented in Table 2. 

In general, organic acids pose another important component of food, as their presence and composition strongly affects the taste of food. Specifically, lactic acid is a natural preservative that besides enhancing the preservation time of foods can act as a flavoring substance, improving the profile of organoleptic characteristics of fermented foods. In addition, the produced lactic acid, especially at 4 weeks of fermentation at 4 °C, proves the existence of fermentation of the juice by the presence of the potentially probiotic strain SP5. Μalic acid, which is considered as the main organic acid of chokeberry, was detected up to 48 h of fermentation and then no amount was detected. The main reason is that the probiotic strain metabolized malic to lactic acid (malolactic fermentation). Indeed, the levels of lactic acid significantly increased during fermentation at 4 °C and remained at high levels during the 4 weeks (>9.0 g/L). Acetic acid concentration was determined in traces after the second week of fermentation at 4 °C and remained at very low concentration during the rest period (0.1 g/L). Acetic acid probably was slightly produced by citric acid, as other researchers have reported in lactic acid fermentation of fruit juices [35]. 

Ethanol did not appear in significant levels as well. No significant content of ethanol was observed during any of the studied time periods. This outcome was mostly expected as lactic acid bacteria are known to metabolize sugar molecules to acids such as lactic and malic acid [7,13]. Because no yeast existed in the initial stage nor was any in the fermented chokeberry juice as a result, no ethanol was produced [26]. 

High sorbitol content has been detected in chokeberry juice by other researchers [36]. More importantly, the increased concentration of sorbitol at all stages of fermentation indicates the quality of the natural raw material used. Another important matter regarding the sugar profile in cultivated black chokeberries is the absence of sucrose, which was also illustrated in the present study and which agrees with the literature [27]. The presence of sucrose in chokeberry-based products suggests the addition of sugar or other fruits and is not welcome by many consumers, wanting their product to be pure. Glucose and fructose were detected in various amounts. It should be underlined that the concentration of the sugars varied. They increased during the first 48 h and then slightly decreased. However, the respective levels for unfermented juice showed that there was an increase in all the studied periods. Researchers have also reported this finding in the past. More specifically, it has been reported that during lactic acid fermentation of various fruit juices the total sugar (sucrose, glucose, and fructose) levels increased significantly during storage. However, this increase was even higher (and statistically significant) in the unfermented juices at the same time [12]. In addition, it has also been reported that unfermented Aronia juice sugars were differentially changed during storage. Specifically, fructose and glucose content increased up to 40–50% from week 1 to week 3, but plateaued at week 4, whereas sorbitol content continued to increase [37]. Likewise, the sugar concentration increased, probably due to polysaccharides hydrolysis through the action of pectinases and other enzymes, as other researchers have proposed [12,38,39].

Regarding all the other chemical parameters determined, there was not any change in chemical composition during the studied period. This verifies that lactic acid fermentation was responsible for the changes that occurred in the fermented juice, such as lactic acid production, depletion of malic acid, and decreased levels of produced sugars compared with unfermented juices.

### 3.3. Total Phenolics and Antioxidant Activity

Total phenol content (TPC) of the fermented chokeberry juice was determined using Folin-Ciocalteu method. The results are presented in Table 3.

Results indicate that the application of lactic acid fermentation through *L. paracasei* SP5 led to a remarkable increase of TPC in all the studied periods compared with the control unfermented sample. A possible explanation of this result is that lactic acid fermentation metabolites produced by the breakdown of anthocyanins and other larger-in-size phenolic compounds led to this increase in TPC, as it was observed in many other cases with other fruit juices (pomegranate, cornelian cherry, etc.) [7,11]. However, it is well established that the applied strains for lactic acid fermentation are critical to the variance of the TPC, as well as the starting substrate. Different LAB strains usually follow many metabolic pathways during fermentation of various fruit juices, leading to the metabolism and biotransformation (mainly due to decarboxylation and reduction reactions) of various phenolic compounds [40]. Likewise, strains have different capacities on the metabolism of phenolics. For instance, fermentation of chokeberry with kefir led to an increase of TPC and to bioaccessible polyphenols [24]. In this study, the effect was positive, leading to increased levels of TPC. It has been reported that various enzymatic activities such as decarboxylase can lead to better biotransformation of phenolics and that *Lactobacillus* strains exhibit this ability [41]. For instance, it has been stated that a *Lactobacillus paracasei* strain was effective in phenolics bioconversions due to its high content of *β*-glucoside and *β*-galactoside [42]. It is noted that more analytical tools can be applied in the near future in order to investigate more accurately the changes in certain groups such as flavonol glycosides, total quercetin, and hydroxybenzoic acids.

**Figure 1:**
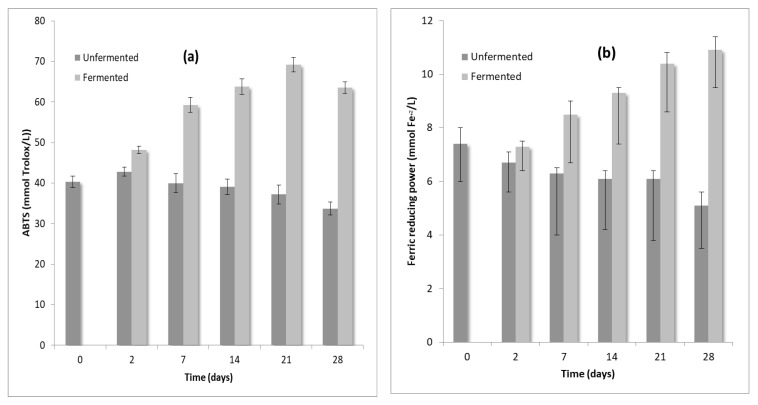
ABTS•+ radical scavenging capacity (**a**) and ferric reducing power (**b**) of unfermented and fermented chokeberry juice at 30 °C for 48 h and during storage (4 °C for 4 weeks).

The changes in the antioxidant activities of unfermented and fermented chokeberry juice with *Lactobacillus paracasei* SP5 during fermentation at 30 °C and at 4 °C were determined through FRAP and αTEAC assays and are presented in Figure 1a,b. These two assays demonstrated that the antioxidant activity of fermented chokeberry juice showed a significant increase mainly during the first week of fermentation at 4 °C, which was observed until the end of the storage (fourth week), compared with the unfermented one. This finding is very important since it seems that the nutritional value of the fermented product was enhanced through fermentation. As similarly noted in the literature the application of potentially probiotic strains applied for juice fermentation may lead to an increase of ABTS radical scavenging activity [29]. In addition, *Lactobacillus* strains may possess high phenolics biotransformation. Likewise, phenolic acids may act as free radical scavengers and possess significant antioxidant activity [43]. Likewise, the increased antioxidant activity of fermented chokeberry juice during fermentation at 4 °C could be attributed: (i) to the ability of *Lactobacillus paracasei* SP5 to provide phenolics biotransformation and (ii) simultaneously to its viability, which was maintained at high levels during storage (above 6 log cfu/mL).

### 3.4. Volatiles Evaluation

The evolution of volatile compounds in chokeberry juices were identified using SPME GC/MS analysis during fermentation of chokeberry juice at 0 h, 48 h, and during fermentation at 4 °C (Table 4). Statistical analysis was performed between the concentrations (μg/g) of each volatile. Alcohols, aldehydes, organic acids, esters, and ketones were the predominant groups of compounds identified in all the time periods, as also detected in most bacterial fermented or unfermented fruit juices [8,10,44]. Most of these volatiles have been identified in chokeberry juices/fruit or other substances fermented by lactic acid bacteria juices or produced through the lactic acid fermentation process. Specifically, 78 compounds were determined in the unfermented juice (28 alcohols, 7 aldehydes, 18 ketones, 7 esters, 6 organic acids, and 12 other compounds), while after 48 h of fermentation 55 compounds were identified (23 alcohols, 4 aldehydes, 13 ketones, 4 esters, 5 organic acids, and 6 other compounds) as presented in Table 4. Esters and alcohols were the major flavor volatile compounds detected in fermented juices during fermentation at 4 °C. It is noteworthy that during the last week (fourth) of fermented juice at 4 °C., 70 compounds were identified (26 alcohols, 3 aldehydes, 17 ketones, 6 esters, 11 organic acids, and 7 other compounds). 

Alcohols, aldehydes, and ketones were the predominant groups determined regarding their total concentration in fermented chokeberry juice, while organic acids surpassed the total concentration of aldehydes in the fourth week of storage. As reported in previous studies, alcohols are important aromatic compounds detected in juices fermented by LAB. In specific, a small content of alcohols can pose a significant effect in the aromatic profile of fruit juices, as they can provide a slight aroma and in parallel pose as solvents for other aromatic volatiles detected in fruit juices [29]. In the present study a significant variety of alcohols was identified at all time periods of fermentation at 4 °C, providing their aromatic effects. More specifically, the volatile compounds determined in chokeberry juice in all time periods included: 3-methyl-1-butanol, 1-hexanol, (2E)-2-hexen-1-ol, benzyl alcohol, phenylethyl alcohol, 2-methylpropan-2-ol, 2-methylbutan-2-ol, 2-methyl-1-propanol (alcohols), 4-methyl-benzenemethanol benzaldehyde, 4-methylbenzaldehyde (aldehydes), 4-methyl-2-pentanone, 3-hexanone, 2-hexanone, 3-penten-2-one, 4-methyl-3-penten-2-one, 5-methyl-3-hexanone, 4-methyl-2-heptanone (ketones), ethyl acetate and methyl benzoate (organic esters), hexanoic acid (organic acids), eucalyptol, and terpinen-4-ol [44,45,46,47,48]. The outcome provided by these identified compounds highlights the stability of the product, as the high content of volatiles was retained even at the fourth week of fermentation at 4 °C (Table 4). 

The occurrence of organic esters during fermentation at 4 °C is considered as a positive fact, as they may pose fruity and floral notes to the fermented juice even at low concentrations [27,48]. Aldehydes on the other hand are considered as unpleasant flavors in foods, while juices may be enhanced in aldehyde content via polyphenols oxidation [51]. For instance, it has been reported that lower aliphatic aldehydes have a strong irritating odor, providing rancid flavors that can have significant effects on fermented liquid products due to their very low threshold value [52]. The decrease of aldehydes’ concentration observed during fermentation at 4 °C of fermented chokeberry juice can be considered as a desirable outcome, improving the final aromatic profile of the juice. 

Terpenes detected in the chokeberry juice can contribute positively to the final aromatic profile, providing pleasant aromatic notes. More specifically, terpenes detected in fermented juice such as D-limonene, eucalyptol, menthol, linalool, terpinen-4-ol, and α-terpineol may contribute to the aromatic profile of fermented juices even at low or trace concentrations due to their very low threshold values. Terpenes were identified in fermented chokeberry juice during the entire storage period, without significant differences in their concentration. As reported in previous studies terpenes can enhance the shelf life of products acting as an antimicrobial [53,54] and antioxidant [55]. Moreover, some terpenes have been identified as having significant health benefits for the consumer, for instance a-terpineol can act against *Helicobacter pylori* or even provide anti-Leishmania activity [56]. The most important outcome at this point is the beneficial effect that terpenes can pose, which were provided constantly as their content did not alter significantly during storage, highlighting the good quality of fermented chokeberry juice. 

## 4. Conclusions

Concerning the outcome of this work, the studied novel *Lactobacillus paracasei* SP5 strain (i) was effective in lactic acid fermentation of chokeberry juice, (ii) preserved its viability in high acidic conditions and even during fermentation at 4 °C for 4 weeks, (iii) led to a product with improved TPC contents and antioxidant activity, and (iv) led to the production of desirable volatile compounds (alcohols and esters) as was proved through the conducted GC-MS analysis. Chokeberry juice seems to be a good substrate for the production of functional beverages of enhanced nutritional value. Furthermore, due to the high astringency of chokeberry juice, lactic acid fermentation with a potentially probiotic strain could be a good way to exploit this fruit and drive it to other products with even better properties and nutritional value. All of the above outcomes, comprise good first steps for facilitating chokeberry juice’s commercial development. However, future work is needed regarding the organoleptic properties of the product (fermented chokeberry juice) and the production of certain metabolites by the *Lactobacillus paracasei* SP5 strain, such as exopolysaccharides and bacteriocins.

## Figures and Tables

**Table 1:** Viability of the potential probiotic strain *Lactobacillus paracasei* SP5 in the fermented chokeberry juice at 30 °C in different initial sugar concentrations (ISC) after 48 h and 4 weeks.

ISC	Time	Cell Counts		
		*L. paracasei* SP5	Yeasts and Fungi	Coliforms
g/L		Log cfu/mL		
61.0	48 h	7.2 ± 0.11 ^c^	nd	nd
	1st week	9.1 ± 0.08 ^a^	nd	nd
	2nd week	9.3 ± 0.13 ^a^	nd	nd
	3rd week	8.2 ± 0.12 ^b^	nd	nd
	4th week	6.5 ± 0.12 ^d^	nd	nd
40.3			nd	nd
	48 h	9.5 ± 0.07 ^b^	nd	nd
	1st week	10.1 ± 0.05 ^a^	nd	nd
	2nd week	10.1 ± 0.12 ^a^	nd	nd
	3rd week	9.1 ± 0.05 ^c^	nd	nd
	4th week	7.9 ± 0.05 ^d^	nd	nd
28.8			nd	nd
	48 h	7.4 ± 0.11 ^c^	nd	nd
	1st week	8.5 ± 0.12 ^b^	nd	nd
	2nd week	9,5 ± 0.10 ^a^	nd	nd
	3rd week	7.5 ± 0.11 ^c^	nd	nd
	4th week	5.1 ± 0.12 ^d^	nd	nd

^a–d^ Different superscript letters in columns for the same ISC indicate statistically significant differences (multifactor (MF)-ANOVA with Tukey’s honestly significant difference (HSD) multiple range test), nd: not detected (no visible colony or less than 10 cfu/mL).

**Table 2:** Analysis of sugars (glucose, fructose, sorbitol), organic acids (malic acid, lactic acid, acetic acid), and ethanol in chokeberry juice with 40.3 g/L initial sugar concentration unfermented (UF) and after fermentation (F) (30 °C for 48 h and 4 °C for 4 weeks).

Time		Μalic Acid	Lactic Acid	Acetic Acid	Glucose	Fructose	Sorbitol	Ethanol
		g/L						(% *v*/*v*)
0 h		4.9 ± 0.18	nd	nd	11.2 ± 0.19	9.1 ± 0.39	20.1 ± 0.20	nd
48 h	UF	4.6 ± 0.19 ^a^	nd	nd	18.3 ± 0.07 ^a^	13.4 ± 0.06 ^a^	24.4 ± 0.15 ^a^	nd
	F	4.1 ± 0.35 ^b^	0.2 ± 0.05	nd	16.1 ± 0.15 ^b^	12.1 ± 0.21 ^b^	24.3 ± 0.18 ^a^	nd
1st week	UF	4.6 ± 0.21	nd	nd	19.1 ± 0.11 ^a^	16.9 ± 0.09 ^a^	22.9 ± 0.05 ^a^	nd
	F	nd	8.9 ± 0.15	nd	15.6 ± 0.19 ^b^	11.8 ± 0.12 ^b^	22.6 ± 0.35 ^a^	nd
2nd week	UF	4.6 ± 0.12	nd	nd	19.9 ± 0.23 ^a^	17.4 ± 0.15 ^a^	21.2 ± 0.41 ^a^	nd
	F	nd	9.1 ± 0.21	0.1 ± 0.02	13.8 ± 0.21 ^b^	10.9 ± 0.08 ^b^	21.1 ± 0.19 ^a^	nd
3rd week	UF	4.5 ± 0.27	nd	nd	22.4 ± 0.31 ^a^	18.3 ± 0.11 ^a^	21.5 ± 0.15 ^a^	nd
	F	nd	9.3 ± 0.39	0.1 ± 0.04	14.0 ± 0.18 ^b^	10.9 ± 0.11 ^d^	21.3 ± 0.24 ^a^	nd
4th week	UF	4.6 ± 0.11	nd	nd	22.9 ± 0.15 ^a^	20.0 ± 0.04 ^a^	22.5 ± 0.45 ^a^	nd
	F	nd	9.4 ± 0.48	0.1 ± 0.05	14.4 ± 0.11 ^b^	11.4 ± 0.15 ^b^	22.2 ± 0.17 ^a^	nd

UF: unfermented juice; F: fermented juice. Different superscript letters in columns indicate statistically significant differences (MF-ANOVA with Tukey’s HSD multiple range test); nd: not detected (<0.1 g/L).

**Table 3:** Total phenolic content (TPC) of the chokeberry juice with 40.3 g/L initial sugar concentration after fermentation (30 °C for 48 h) and during storage (4 °C for 4 weeks).

Time	Chokeberry Juice Samples	TPC GAE g/L
0 h	Unfermented	8.5 ± 0.1
	Fermented	
48 h	Unfermented	8.6 ± 0.2 ^b^
	Fermented	9.8 ± 0.2 ^a^
1st week	Unfermented	8.9 ± 0.3 ^b^
	Fermented	11.2 ± 0.3 ^a^
2nd week	Unfermented	9.0 ± 0.1 ^b^
	Fermented	11.9 ± 0.1 ^a^
3rd week	Unfermented	9.0 ± 0.3 ^b^
	Fermented	12.0 ± 0.2 ^a^
4th week	Unfermented	8.1 ± 0.2 ^b^
	Fermented	12.1 ± 0.2 ^a^

^a,b^ Different superscript letters in columns (at the same time between fermented and unfermented juice) indicate statistically significant differences (MF-ANOVA with Tukey’s HSD multiple range test).

**Table 4:** Volatile compounds identified in the fermented chokeberry juice at 0 h, 48 h, and during fermentation at 4 °C.

Compound	Identification ^1^	RI	Content (μg/g) ^2^				Reference
			0 h	48 h	1st Week	4th Week	
Alcohols							
Ethanol	A	932	10.0 ± 2.23 ^d^	55.0 ± 3.47 ^c^	69.0 ± 4.71 ^b^	99.0 ± 5.35 ^a^	[45]
3-Methyl-1-butanol	A	1213	6.3 ± 1.33 ^a^	2.5 ± 0.77 ^b^	1.7 ± 1.05 ^b^	1.6 ± 0.93 ^b^	[45]
2-Ethyl-1-hexanol	A	1494	0.4 ± 0.05 ^b^	0.2 ± 0.05 ^c^	0.4 ± 0.08 ^b^	0.6 ± 0.03 ^a^	[45]
1-Penten-3-ol	B	1165	0.2 ± 0.04	tr	tr	tr	[45]
1-Hexanol	A	1358	18.8 ± 3.14 ^a^	1.7 ± 0.05 ^c^	2.0 ± 0.10 ^b^	1.9 ± 0.05 ^b^	[45]
(3E)-3-Hexen-1-ol	B	1367	0.1 ± 0.03 ^a^	0.1 ± 0.04 ^a^	0.1 ± 0.04 ^a^	0.1 ± 0.03 ^a^	[46]
(3Z)-3-Hexen-1-ol	B	1387	0.7 ± 0.14	tr	tr	tr	[46]
(2E)-2-Hexen-1-ol	B	1409	3.7 ± 0.39 ^a^	0.1 ± 0.04 ^d^	0.3 ± 0.04 ^b^	0.2 ± 0.03 ^c^	[46]
(2Z)-2-Penten-1-ol	B	1316	0.1 ± 0.04 ^a^	0.1 ± 0.03 ^a^	tr	tr	[45]
Benzyl alcohol	B	1875	35.3 ± 4.81 ^a^	0.3 ± 0.05 ^a^	0.1 ± 0.04 ^c^	0.1 ± 0.04 ^c^	[45]
Phenylethyl Alcohol	A	1912	1.2 ± 0.16 ^a^	0.2 ± 0.05 ^b^	0.1 ± 0.02 ^c^	0.1 ± 0.03 ^c^	[45]
2-Methylpropan-2-ol	B	907	10.1 ± 2.09 ^a^	4.1 ± 0.89 ^b^	4.8 ± 1.34 ^b^	4.8 ± 1.07 ^b^	
2-Methylbutan-2-ol	B	1018	3.9 ± 0.63 ^a^	1.7 ± 0.35 ^c^	1.9 ± 0.27 ^c^	2.8 ± 0.34 ^b^	
1-Propanol	A	1043	0.3 ± 0.05	tr	tr	tr	
2-Methyl-3-buten-2-ol	B	1045	tr	tr	0.1 ± 0.02 ^a^	0.1 ± 0.06 ^a^	
2-Methyl-1-propanol	A	1103	5.4 ± 1.23 ^a^	1.9 ± 0.47 ^b^	1.4 ± 0.38 ^b^	1.6 ± 0.34 ^b^	[47]
2-Methylpentan-2-ol	B	1113	0.8 ± 0.02 ^a^	0.4 ± 0.03 ^c^	0.5 ± 0.02 ^b^	0.5 ± 0.01 ^b^	[47]
3-Pentanol	A	1117	0.1 ± 0.04	tr	tr	tr	
2-Pentanol	A	1129	3.3 ± 0.04 ^a^	0.6 ± 0.05 ^b^	0.3 ± 0.04 ^c^	0.3 ± 0.07 ^c^	[48]
1-Butanol	A	1151	0.4 ± 0.07 ^a^	0.1 ± 0.04 ^b^	0.1 ± 0.03 ^b^	tr	[48]
4-Methyl-2-pentanol	B	1174	0.2 ± 0.04 ^a^	0.1 ± 0.03 ^b^	0.1 ± 0.03 ^b^	0.2 ± 0.05 ^a^	
3-Methyl-3-buten-1-ol	B	1251	1.5 ± 0.05 ^b^	0.6 ± 0.02 ^c^	1.8 ± 0.08 ^a^	2.0 ± 0.14 ^a^	
1-Pentanol	A	1255	1.5 ± 0.05 ^a^	0.3 ± 0.03 ^b^	0.3 ± 0.04 ^b^	0.3 ± 0.04 ^b^	[48]
3-Methyl-2-buten-1-ol	C	1324	0.7 ± 0.05 ^b^	0.1 ± 0.04 ^c^	0.9 ± 0.10 ^a^	0.6 ± 0.10 ^b^	
1-Octen-3-ol	A	1454	0.3 ± 0.05 ^a^	0.1 ± 0.04 ^b^	0.1 ± 0.03 ^b^	0.1 ± 0.04 ^b^	[48]
(2E)-2-Hepten-1-ol	C	1514	0.1 ± 0.02	tr	tr	tr	
1-Octanol	A	1562	0.4 ± 0.05 ^a^	0.1 ± 0.01 ^c^	0.3 ± 0.03 ^b^	0.4 ± 0.05 ^a^	[48]
(2E)-2-Octen-1-ol	C	1619	tr	tr	0.1 ± 0.04 ^b^	0.2 ± 0.05 ^a^	
1-Nonanol	B	1665	0.1 ± 0.05 ^a^	tr	tr	0.1 ± 0.03 ^a^	
2-Nonanol	B	1525	tr	tr	0.4 ± 0.05 ^a^	0.2 ± 0.04 ^b^	
4-Methyl-benzenemethanol	B	1961	3.8 ± 1.29 ^a^	0.4 ± 0.05 ^b^	tr	0.1 ± 0.04 ^c^	
1-Dodecanol	B	1972	tr	0.2 ± 0.05 ^c^	0.4 ± 0.07 ^b^	0.7 ± 0.05 ^a^	
4-Methyl-2-heptanol	C	1366	tr	tr	0.1 ± 0.05 ^a^	0.1 ± 0.04 ^a^	
Aldehydes							
Acetaldehyde	A	704	1.8 ± 0.72 ^a^	0.3 ± 0.05 ^b^	tr	tr	[45]
Hexanal	A	1077	0.4 ± 0.08	tr	tr	tr	[45]
Nonanal	B	1391	0.1 ± 0.03	tr	tr	tr	[46]
(2E)-2-Octenal	C	1419	0.1 ± 0.03	tr	tr	tr	[48]
Benzaldehyde	A	1517	11.2 ± 0.63 ^a^	7.6 ± 0.95 ^b^	0.5 ± 0.05 ^d^	0.9 ± 0.10 ^c^	[46]
4-Methylbenzaldehyde	B	1643	14.9 ± 1.92 ^a^	5.8 ± 1.04 ^b^	6.4 ± 1.36 ^b^	6.8 ± 1.47 ^b^	
3-Methylbenzaldehyde	B	1612	0.2 ± 0.07 ^a^	0.3 ± 0.10 ^a^	0.2 ± 0.04 ^a^	0.2 ± 0.05 ^a^	
Ketones							
2-Butanone	B	901	4.1 ± 0.76 ^a^	1.8 ± 0.17 ^c^	2.2 ± 0.14 ^b^	2.2 ± 0.17 ^b^	[48]
2,3-Butanedione	A	970	14.2 ± 3.71 ^a^	5.0 ± 0.97 ^c^	6.3 ± 0.86 ^c^	9.3 ± 1.09 ^b^	[48]
4-Methyl-2-pentanone	B	1005	0.8 ± 0.05 ^a^	0.5 ± 0.03 ^c^	0.6 ± 0.04 ^b^	0.6 ± 0.04 ^b^	
3-Hexanone	B	1048	0.1 ± 0.04 ^a^	0.1 ± 0.03 ^a^	0.1 ± 0.04 ^a^	0.1 ± 0.02 ^a^	
2-Hexanone	B	1076	0.5 ± 0.14 ^a^	0.3 ± 0.11 ^a^	0.3 ± 0.13 ^a^	0.3 ± 0.15 ^a^	
3-Penten-2-one	B	1123	28.3 ± 3.08 ^a^	6.5 ± 1.83 ^c^	10.7 ± 1.73 ^b^	10.1 ± 1.95 ^b^	[45]
4-Methyl-3-penten-2-one	B	1128	0.2 ± 0.05 ^b^	0.7 ± 0.09 ^a^	0.1 ± 0.07 ^b^	0.2 ± 0.04 ^b^	[45]
5-Methyl-3-hexanone	C	1149	0.1 ± 0.03 ^a^	tr	tr	0.1 ± 0.03 ^a^	
2-Heptanone	B	1178	0.2 ± 0.04 ^b^	0.1 ± 0.02 ^c^	0.5 ± 0.05 ^a^	0.3 ± 0.10 ^b^	[48]
4-Methyl-2-heptanone	B	1203	1.3 ± 0.17 ^b^	0.6 ± 0.13 ^c^	1.7 ± 0.11 ^a^	1.7 ± 0.14 ^a^	
1-Hydroxy-2-propanone	B	1294	tr	tr	0.1 ± 0.03 ^a^	0.1 ± 0.04 ^a^	
5-Methyl-3-hexen-2-one	C	1230	0.1 ± 0.03 ^a^	tr	0.1 ± 0.03 ^a^	0.1 ± 0.02 ^a^	
4,6-Dimethyl-2-heptanone	C	1241	0.1 ± 0.04 ^b^	0.1 ± 0.02 ^b^	0.2 ± 0.03 ^a^	0.2 ± 0.03 ^a^	
6-Methyl-5-hepten-2-one	C	1335	0.4 ± 0.11 ^a^	0.1 ± 0.09 ^b^	tr	tr	[46]
2,7-Octanedione	C	1342	0.1 ± 0.02 ^b^	tr	0.2 ± 0.05 ^a^	0.2 ± 0.05 ^a^	
2-Nonanone	B	1387	tr	tr	0.1 ± 0.03	tr	[48]
2,5-Hexanedione	B	1500	0.4 ± 0.05 ^a^	0.1 ± 0.03 ^b^	0.1 ± 0.04 ^b^	0.1 ± 0.04 ^b^	
2,3-Butanediol isomer 1	C	1544	0.2 ± 0.03	tr	tr	tr	
4-Ethyl-1,3-benzenediol	C	1572	0.5 ± 0.03 ^a^	0.2 ± 0.02 ^c^	0.3 ± 0.04 ^b^	0.3 ± 0.05 ^b^	
(5E)-6,10-Dimethyl-5,9-undecadien-2-one	C	1854	0.1 ± 0.03 ^a^	tr	0.1 ± 0.02 ^a^	0.1 ± 0.04 ^a^	
Esters							
Ethyl Acetate	A	884	5.9 ± 1.19 ^a^	0.3 ± 0.04 ^b^	0.2 ± 0.03 ^c^	0.4 ± 0.08 ^b^	[45]
2-Methylpropyl formate	B	959	0.2 ± 0.10 ^a^	0.3 ± 0.10 ^a^	0.3 ± 0.14 ^a^	0.3 ± 0.11 ^a^	
Isobutyl acetate	A	1012	0.1 ± 0.02 ^b^	0.2 ± 0.04 ^a^	0.2 ± 0.04 ^a^	0.2 ± 0.05 ^a^	
Ethyl 2-hydroxypropanoate	A	1344	tr	tr	0.1 ± 0.03 ^b^	0.3 ± 0.04 ^a^	
Octyl octanoate	B	2013	0.5 ± 0.17 ^a^	tr	0.1 ± 0.03 ^b^	0.1 ± 0.04 ^b^	
Methyl benzoate	B	1618	1.6 ± 0.14 ^a^	1.0 ± 0.10 ^b^	1.5 ± 0.21 ^a^	1.7 ± 0.15 ^a^	[48]
Ethyl benzoate	B	1664	0.3 ± 0.05	tr	tr	tr	[48]
2-Phenethyl acetate	A	1811	0.2 ± 0.04	tr	tr	tr	[49]
Organic acids							
Acetic acid	A	1446	0.8 ± 0.31 ^c^	1.0 ± 0.27 ^c^	4.6 ± 0.15 ^a^	4.2 ± 0.13 ^b^	[45,50]
2-Methylpropanoic acid	C	1568	0.1 ± 0.03 ^a^	0.1 ± 0.02 ^a^	0.1 ± 0.04 ^a^	0.1 ± 0.03 ^a^	
Butanoic acid	B	1628	tr	tr	0.1 ± 0.03 ^a^	0.1 ± 0.03 ^a^	[50]
3-Methyl-butanoic acid	B	1670	tr	0.2 ± 0.05 ^a^	0.2 ± 0.05 ^a^	0.2 ± 0.04 ^a^	
2-Methyl-butanoic acid	C	1672	0.5 ± 0.12 ^a^	0.2 ± 0.04 ^b^	0.2 ± 0.06 ^b^	0.2 ± 0.04 ^b^	[45]
Heptanoic acid	B	1954	0.1 ± 0.04 ^a^	tr	0.1 ± 0.03 ^a^	0.1 ± 0.04 ^a^	[49]
Hexanoic acid	A	1844	0.6 ± 0.04 ^a^	0.3 ± 0.09 ^b^	0.4 ± 0.08 ^b^	0.5 ± 0.04 ^b^	[46]
Octanoic acid	A	2062	tr	tr	tr	0.6 ± 0.10	[46]
Nonanoic acid	C	2174	0.1 ± 0.04 ^b^	tr	tr	0.3 ± 0.05 ^a^	[46]
2-Hydroxy-2-methylmalonic acid	C	2183	tr	tr	3.6 ± 1.02 ^a^	1.0 ± 0.47 ^b^	
n-Decanoic acid	Β	2250	tr	tr	tr	0.1 ± 0.02	[46]
Others							
D-Limonene	A	1186	0.1 ± 0.04	tr	tr	tr	[46]
Eucalyptol	B	1200	1.8 ± 0.22 ^a^	0.2 ± 0.07 ^c^	0.6 ± 0.05 ^b^	0.7 ± 0.09 ^b^	[45]
p-Cymene	B	1263	0.1 ± 0.03	tr	tr	tr	[46]
Acetoin	A	1281	0.4 ± 0.05 ^c^	0.2 ± 0.05 ^d^	0.7 ± 0.08 ^b^	1.1 ± 0.19 ^a^	[48]
cis-Linalool oxide	B	1445	0.1 ± 0.04	tr	tr	tr	[46]
Linalool	A	1551	0.1 ± 0.04 ^b^	tr	0.1 ± 0.03 ^b^	0.2 ± 0.05 ^a^	[48]
Terpinen-4-ol	B	1604	2.7 ± 0.61 ^a^	0.4 ± 0.09 ^b^	tr	tr	[48,49]
Menthol	B	1644	0.1 ± 0.03 ^b^	tr	0.2 ± 0.05 ^a^	0.2 ± 0.05 ^a^	[48]
α-Terpineol	A	1699	0.1 ± 0.03	tr	tr	tr	[45]
β-Damascenone	C	1818	0.2 ± 0.05 ^a^	0.2 ± 0.05 ^a^	0.2 ± 0.05 ^a^	0.2 ± 0.07 ^a^	[46]
5-Methyl-3-methylenedihydro-2(3H)-furanone	C	1827	1.2 ± 0.14 ^a^	0.5 ± 0.05 ^d^	0.7 ± 0.04 ^c^	0.9 ± 0.08 ^b^	[50]
Geraniol	A	1849	0.2 ± 0.07 ^a^	0.3 ± 0.05 ^a^	0.2 ± 0.05 ^a^	0.2 ± 0.03 ^a^	[46]

^1^ Identification: A-level: agreement of retention index (RI) and mass spectrum (MS) with those of an authentic compound analyzed under identical experimental conditions; B-level: agreement of retention index (ΔRI < 20) and mass spectrum (match > 900); C-level: at least ΔRI < 20 or mass spectrum similarity match > 800. ^2^ The content of individual components was calculated relative to the internal standard and expressed as μg/g juice. All calculations were based on peak area (from AMDIS). Different superscript letters within rows indicate statistically significant differences (MF-ANOVA with Tukey’s HSD multiple range test), tr: traces (<0.1 μg/g).
